# Hypoxia-inducible factor signaling in the development of kidney fibrosis

**DOI:** 10.1186/1755-1536-5-S1-S16

**Published:** 2012-06-06

**Authors:** Volker H Haase

**Affiliations:** 1 Division of Nephrology and Hypertension, Departments of Medicine, Molecular Physiology and Biophysics, and Cancer Biology, Vanderbilt School of Medicine, Nashville, TN, USA

## Abstract

A discrepancy between oxygen availability and demand has been found in most chronic kidney diseases (CKD) irrespective of etiology. This results from a combination of structural and functional changes that are commonly associated with the development of fibrosis, which include a reduction in peritubular blood flow, luminal narrowing of atherosclerotic vessels, capillary rarefaction and vascular constriction due to altered expression of vasoactive factors and signaling molecules (e.g. angiotensin II, endothelin, nitric oxide). Consistent with decreased renal oxygenation in CKD is the increased expression of the oxygen-sensitive α-subunit of hypoxia-inducible factor (HIF)-1. HIF transcription factors are members of the Per-ARNT-Sim (PAS) family of heterodimeric basic helix-loop-helix transcription factors and consist of an oxygen-sensitive α-subunit and a constitutively expressed β-unit, also known as the aryl-hydrocarbon-receptor nuclear translocator (ARNT) or HIF-β. Recent experimental evidence suggests that prolonged activation of HIF signaling in renal epithelial cells enhances maladaptive responses, which lead to fibrosis and further tissue destruction. Cell type-specific functions of individual HIF transcription factors and their relevant transcriptional targets are discussed in the context of renal fibrogenesis.

## Introduction

Despite the very large blood flow (~20% of total cardiac output), the kidneys, which carry out complex and energy consuming cellular transport functions, operate under markedly reduced oxygen tension, with regional oxygen levels ranging from 10 to 60 mmHg. A reduction in renal oxygenation occurs in most chronic kidney diseases (CKD) irrespective of etiology. This is due to a combination of several pathophysiological and morphologic changes, which are typically associated with chronic kidney injury. These include increased oxygen demand from hyperfiltration and tubular hypertrophy, capillary rarefaction, glomerular injury, luminal narrowing of atherosclerotic vessels, as well as vascular constriction due to altered expression of vasoactive factors and signaling molecules (e.g. angiotensin II, endothelin, nitric oxide). The resulting reduction in renal oxygen availability is furthermore exacerbated by extra-cellular matrix (ECM) expansion, which limits oxygen diffusion, and by renal anemia [1,2]. Blood oxygen level-dependent (BOLD) MRI, molecular and histological techniques, as well as measurements of renal oxygen levels with microelectrodes have been used to assess tissue oxygenation in chronic kidney diseases, including diabetic and IgA nephropathy, obstructive nephropathy, fibrosis associated with 5/6 nephrectomy and anti-Thy1 glomerulonephritis (for an overview of these studies see [1-4]).

Consistent with decreased renal oxygenation in CKD is the increased expression of the oxygen-sensitive α-subunit of hypoxia-inducible factor (HIF)-1 in renal biopsy material from patients with CKD [5,6]. The heterodimeric basic helix-loop-helix transcription factors HIF-1 and HIF-2 are key mediators of cellular adaptation to hypoxia, and belong to the PAS {PER/aryl-hydrocarbon-receptor nuclear translocator (ARNT)/single minded (SIM)} family of transcription factors. They consist of an oxygen-sensitive α-subunit and a constitutively expressed β-subunit, which is also known as the aryl hydrocarbon receptor nuclear translocator (ARNT), and facilitate both oxygen delivery and cell survival by stimulating erythropoiesis, angiogenesis and anaerobic energy metabolism. HIF-1 and HIF-2 are furthermore involved in the regulation of biological processes that are relevant to wound healing, tissue repair and fibrogenesis, such as extracellular matrix synthesis and turnover, cell adhesion and migration, and epithelial to mesenchymal transition (EMT) [7-12].

**Figure 1:**
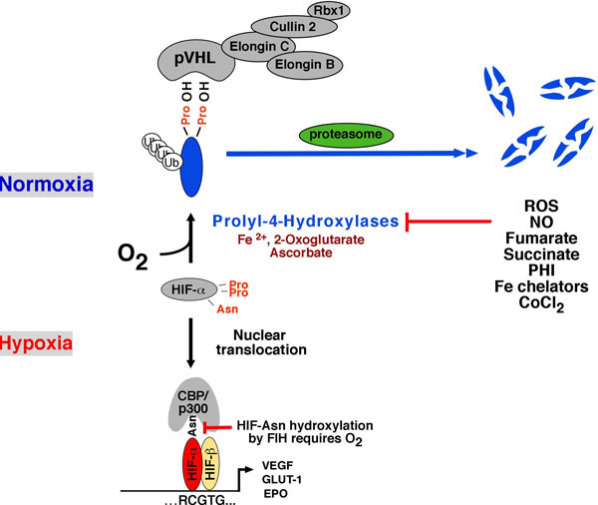
**Overview of PHD/HIF signaling**. Under normoxia, both HIF-1α and HIF-2α are hydroxylated by prolyl-4-hydroxylases and are targeted for proteasomal degradation by the von Hippel-Lindau (pVHL)-E3 ubiquitin ligase complex (shown are key components of this complex). Binding to prolyl-hydroxylated HIF-α occurs at the β-domain of pVHL, which spans amino acid residues 64 - 154. The C-terminal α-domain links the substrate recognition component pVHL to the E3 ubiquitin ligase via elongin C. When prolyl-4-hydroxylation is inhibited (e.g. by hypoxia, ROS), HIF-α subunits are stabilized and translocate to the nucleus where they heterodimerize with ARNT. HIF-α/ARNT heterodimers bind to the HIF consensus-binding site, RCGTG, resulting in increased expression of target genes. Factor-inhibiting-HIF (FIH) is a dioxygenase that modulates transcriptional cofactor recruitment (CBP/p300) via asparagine (Asn) hydroxylation of the HIF-α carboxy-terminal transactivation domain. In addition to ROS, nitric oxide, Krebs cycle metabolites succinate and fumarate, cobalt chloride and iron chelators such as desferrioxamine inhibit HIF prolyl-4-hydroxylases in the presence of oxygen. **Abb.: **CoCl_2_, cobalt chloride; Fe^2+^, ferrous iron; NO, nitric oxide; PHI, prolyl-4-hydroxylase inhibitors (structural 2-oxoglutarate analogs); ROS, reactive oxygen species; ub, ubiquitin.

HIF heterodimers activate gene transcription in response to hypoxia by binding to specific DNA sequences, which are known as hypoxia-response elements (HREs) and by recruiting transcriptional co-activators such as CBP/p300 (Figure 1). While HIF-α is continuously synthesized, it is rapidly degraded under normoxia, keeping HIF signaling at minimal levels when oxygen tension is in normal range. HIF degradation under normoxia requires hydroxylation of specific proline residues within the oxygen-dependent degradation domain of HIF-α, enabling interaction with the von Hippel-Lindau tumor suppressor pVHL, which functions as the substrate recognition component of an E3 ubiquitin ligase complex [13,14]. HIF hydroxylation depends on the presence of molecular oxygen, ferrous iron and ascorbate, and is carried out by 2-oxoglutarate-dependent dioxygenases (prolyl-4-hydroxylase domain (PHD) proteins). Three major HIF-hydroxylating enzymes have been identified, PHD1, 2 and 3, of which PHD2 is most important for normoxic HIF degradation [14]. A second hypoxic switch operates in the carboxy-terminal transactivation domain of HIF-α with the hydroxylation of an asparagine residue. Under hypoxic conditions asparagine hydroxylation is inhibited and CBP/p300 recruitment facilitated, enabling increased levels of transcription [14].

While HIF has been shown to be cytoprotective in acute kidney injury [3], work by our laboratory and others now suggests that prolonged activation of HIF signaling in renal epithelial cells enhances maladaptive responses, which lead to fibrosis and tissue destruction. Specifically, our laboratory has identified epithelial HIF-1α as a promoter of kidney fibrosis, and has demonstrated that HIF-1 activation stimulates collagen accumulation and inflammatory cell recruitment in experimental models of CKD [5,15].

## Results

### Evidence of HIF activation in renal biopsy material from patients with CKD

**Figure 2:**
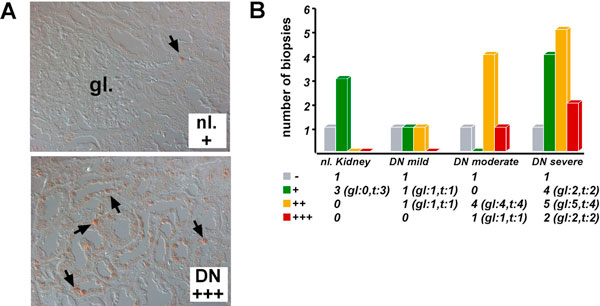
**HIF-1α expression in diabetic nephropathy**. HIF-1α immunostaining in formalin-fixed, paraffin-embedded renal biopsy tissues from patients with diabetic nephropathy (DN). **(A) **Shown are non-affected control kidney tissue (nl. +, less than 25% of tubular epithelial cells are stained) and kidney tissue from a patient with severe DN (DN +++, positive staining is detected in >50% of tubular epithelial cells). Arrows highlight cells with nuclear HIF-1α staining. **(B) **Summary of HIF-1α expression analysis in DN. DN cases are grouped according to tubulointerstitial injury score as previously described [53]. The number of biopsies with glomerular (gl) or tubular staining (t) is shown in brackets. -, absence of staining; +: 1-25% of cells per visual field with staining; ++: >25-50% and +++: >50% of cells with positive staining.

**Figure 3:**
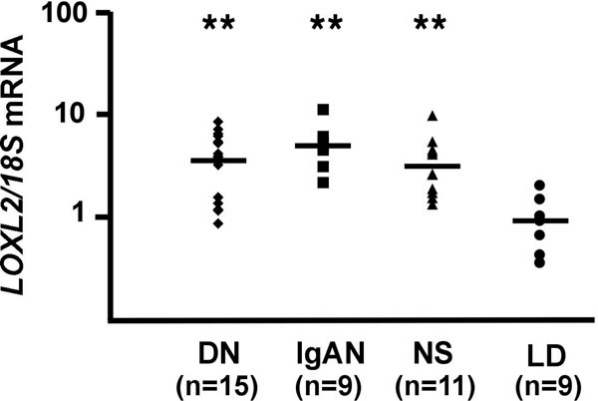
**Increased expression of *LOXL2 *in CKD**. Real time PCR analysis of *LOXL2 *expression in micro-dissected tubulointerstitium from patients with DN, IgA nephropathy (IgAN) and hypertensive nephrosclerosis (NS). Shown are relative expression values normalized to *18S*. Pre-transplant biopsies from living related donor kidneys (LD) were used as control. ** P < 0.001 by Mann-Whitney test.

A discrepancy between oxygen availability and demand has been demonstrated in experimental CKD. In order to investigate the expression levels of HIF-1α in tissues from patients with CKD, we have used immunohistochemistry to analyze archival, paraffin-embedded renal biopsy material from patients with different stages of diabetic nephropathy, which is the leading cause of end stage renal disease. We found a statistically significant correlation of disease stage with the percentage of HIF-1α-expressing tubular epithelial cells, which suggested that the level of hypoxia in diabetic CKD associates with disease severity, extent of fibrosis and disease progression (Figure 2) [5]. HIF-1α was also detected in fibrotic areas of renal tissues from patients with IgA nephropathy [5]. Genome-wide gene expression analysis of micro-dissected renal biopsy material from patients with diabetic nephropathy (tubulointerstitium only) revealed that approximately 50 of 1349 differentially regulated genes were established or putative HIF transcriptional targets [5]. Increased expression was found for *phosphoglycerate kinase-1 (PGK-1)*, chemokine receptor *CXCR4*, *lysyl oxidase-like 2 *(*LOXL2*) and *phosphofructokinase *(*PFKFB3*). A comparable increase in *LOXL2 *expression was also seen by real-time PCR in micro-dissected tubulointerstitium from patients with IgA nephropathy and hypertensive nephrosclerosis (Figure 3), suggesting a general association between *LOXL2 *expression and the presence of CKD [5]. Taken together with studies in experimental animal models, our clinical findings provide strong evidence that activation of the HIF system associates with the development and progression of renal fibrosis.

### HIF promotes EMT in primary renal epithelial cells

Recent findings by our laboratory and other groups suggest that hypoxia, via activation of HIF-1 signaling, enhances fibrogenesis through HRE-mediated upregulation of extracellular matrix (ECM) modulators, such connective tissue growth factor (CTGF), tissue inhibitor of matrix metalloproteinase (TIMP)-1 and plasminogen activator inhibitor (PAI)-1 [16-18]. PAI-1, for example, is known to enhance fibrosis when overexpressed in transgenic mice, whereas PAI-1 deficiency is protective [19,20].

**Figure 4:**
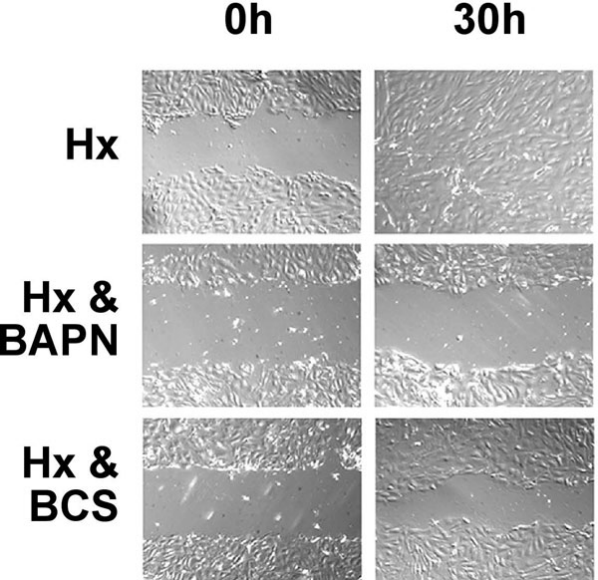
**LOX inhibition impairs migration of primary renal epithelial cells in vitro**. Cells were cultured under hypoxia for 6 days, 2 h prior to placing a scratch wound, lysyl oxidase inhibitors BAPN or BCS were added and cell migration monitored over 30 h. Hx: Control cells (hypoxia without inhibitor). Magnification ×100.

An important cellular process, which is associated with the development of tubulointerstitial fibrosis, is EMT [21-23]. EMT in the setting of CKD is considered a disease-promoting process by which epithelial cells acquire a mesenchymal phenotype and then migrate into the interstitial compartment through breaks in the basement membrane, where they together with resident cells produce extracellular matrix as myofibroblasts [24]. To explore whether EMT in renal tubular epithelial cells is modulated by hypoxia via HIF, we isolated primary tubular epithelial cells (PTECs) from the kidney cortex of genetically modified mice, which express a tetracycline-inducible Cre-recombinase permitting inactivation of HIF-1α in vitro. Using this approach, we demonstrated that hypoxia (1% O_2_) led to morphologic and gene expression changes that were consistent with EMT, and furthermore enhanced the migratory ability of PTECs in a HIF-1-dependent fashion. Since HIF-regulation of lysyl oxidase (LOX) was required for hypoxia-induced migration of breast cancer cells [25], we investigated whether lysyl oxidases were involved in mediating the EMT-promoting effects of hypoxia in PTECs. We first established that the hypoxic regulation of *LOX *and its homologue *LOXL2 *was HIF-1-dependent, and then used pharmacological means to investigate the effects of lysyl oxidase inhibition on epithelial cell migration. Lysyl oxidase inhibitors β-aminoproprionitrile (BAPN) or bathocuproine disulphate (BCS) phenocopied the effects of HIF-1 inactivation, and attenuated hypoxia-enhanced migration in a scratch wound closure assay; complete closure was observed by 24 hours in hypoxic cells, whereas pre-treatment with either BAPN or BCS inhibited migration with the scratch not closing for at least 30 hours (Figure 4). Lysyl oxidases are copper-dependent enzymes with intracellular and extracellular activities, and catalyze the oxidation of lysine residues in collagen and elastin fibers, thereby modulating cell migration and epithelial differentiation [26,27]. BAPN is an irreversible inhibitor of lysyl oxidases and inhibits all lysyl oxidase family members.

Taken together, our data suggested that hypoxia induces morphologic and molecular changes that are consistent with EMT. Although some changes did not involve HIF-1, e.g. increased expression of α-smooth muscle actin (SMA), augmentation of epithelial migration in hypoxia involved HIF-1-mediated induction of lysyl oxidases.

### Inactivation of epithelial HIF-1 improves renal fibrosis in vivo

To investigate the contribution of epithelial HIF-1 signaling to the development of kidney fibrosis in vivo, we used a model of renal fibrogenesis that is based on unilateral ureteral obstruction (UUO). In this model one of the ureters is ligated, blocking urine flow completely, which results in rapidly progressing fibrosis in the affected kidney. UUO kidneys develop hypoxia, as demonstrated by pimonidazole staining and epithelial HIF-1α stabilization. HIF-2α was detected in tubulointerstitial cells, which is consistent with immunohistochemical studies in hypoxic kidneys [3,5].

To assess the contribution of epithelial HIF-1 to fibrosis in this model, we compared wild type mice to animals that were HIF-1-defcient in the proximal renal tubule. Proximal tubule-restricted inactivation of HIF-1 was accomplished through use of a Cre-recombinase under the control of the phosphoenolpyruvate carboxykinase (PEPCK) promoter. Inactivation of HIF-1 in proximal tubule epithelial cells resulted in reduced accumulation of collagen, reduced macrophage accumulation and reduced expression of FSP-1-positive cells following UUO [5], supporting our in vitro findings and the notion that HIF-1 functions as a profibrotic transcription factor under chronic injury conditions.

### Increased expression of epithelial HIF promotes fibrosis in vivo

Since genetic inactivation of HIF-1α in the context of UUO resulted in attenuation of fibrosis and inflammation, we next investigated whether activation of epithelial HIF in a normoxic context could enhance fibrogenesis in vivo. For these studies we used Cre-recombinase transgenes that either were driven by the gamma-glutamyl transpeptidase (γ-GT) promoter or were tetracycline-inducible under the control of a Pax8-driven transactivator [28]. Inhibition of HIF-α degradation as a result of pVHL inactivation produced inflammation and rapid extracellular matrix deposition in the medulla (Liu, Kobayashi, Farsijani and Haase, unpublished data). HIF activation in the proximal tubule accelerated fibrosis in a CKD model based on 5/6 nephrectomy, and resulted in spontaneous fibrosis development in older mice [15]. Pharmacologic inhibition of HIF-1 with YC-1 compound [3-(5'-hydroxymethyl-2'-furyl)-1-benzylindazole] abrogated these effects and slowed the development of fibrosis in mice subjected to UUO [15], suggesting that HIF-1 may represent a novel pharmacologic target to slow the progression of CKD.

## Discussion

Renal epithelial cells contribute to the development of kidney fibrosis, as they increase and remodel ECM when stimulated with TGF-β1, angiotensin II and other cytokines [29,30], or when they transition into myofibroblasts as a result of EMT [23,24]. Increased HIF expression has been found in animal models of CKD and in renal biopsy material from patients with diabetic nephropathy and other forms of renal disease, where it correlates with disease severity [5,31,32]. Our laboratory has identified epithelial HIF-1 as a promoter of renal fibrosis in experimental UUO [5]. The biological outcome of activated HIF signaling, however, is different under conditions of acute renal hypoxia [3]. HIF-1α can be detected in the nucleus of renal tubular epithelial cells, where it dimerizes with HIF-1β to form transcriptionally active HIF-1. Whereas HIF-1α is expressed in renal epithelial and endothelial cells, HIF-2α is expressed in erythropoietin (EPO)-producing renal interstitial fibroblasts, endothelial and glomerular cells [3]. Genetic studies in mice have demonstrated that reduced expression of either HIF-1α or HIF-2α worsens clinical outcome of acute ischemic kidney injury [33,34]. While the cell types that contribute to HIF-mediated cytoprotection remain to be determined, endothelial HIF-2 appears to ameliorate renal ischemia reperfusion injury through increased expression of ROS scavenging enzymes, such as superoxide dismutase (SOD) [33].

In the context of chronic renal injury the biological consequences of HIF activation are different, and negatively impact clinical outcome, that is, promote fibrosis, ultimately leading to the development of end stage renal disease. This may occur through increased expression of i) ECM-modifying genes, such as LOX and PAI-1, ii) functional co-operation with transforming growth factor (TGF)-β1, iii) promotion of epithelial to mesenchymal transition (EMT), and iv) through modulation of renal inflammation [35]. Hypoxia induces *collagen I*, decreases *matrix-metallopeptidase 2 *(*MMP-2*) in renal epithelial cells [36], and increases *PAI-1 *[18], *tissue-inhibitor of metalloproteinase-1 *(*TIMP-1*) [36] as well as *connective tissue growth factor *(*CTGF*) [16] through HIF-mediated transcriptional responses. Synergistic co-operation between HIF and non-HIF pathways, such as the pro-fibrotic TGF-β1/SMAD3 signaling pathway, may further enhance the expression of HIF-regulated genes in the CKD setting; HIF-1 and TGF-β1 have been shown to co-regulate *vascular endothelial growth factor *(*VEGF*) [37], *endoglin *[38] and *EPO *[39]. The notion of HIF and TGF-β1 working together in the context of CKD is furthermore supported by the observation that hypoxia synergizes with TGF-β1 with regard to the production of certain collagens [40,41].

While hypoxia is the main stimulus for HIF activation, signaling molecules with key roles in the pathogenesis of CKD, such as angiotensin II (Ang II), have also been shown to activate HIF-1 via prolyl-4-hydroxylase inhibition. PHD inhibition in this setting results from increased ROS generation and diminished intracellular ascorbate levels, leading to the oxidation of a critical iron atom that is positioned in the center of the PHD catalytic domain [42]. Although AngII-generated ROS is largely derived from activation of NADPH oxidase [43], others have suggested that AngII-induced HIF-1α stabilization may be mediated by mitochondrial ROS [44]. In this context, recent studies in renal interstitial fibroblasts have proposed that HIF-1 functions downstream of an Ang II-induced pro-fibrotic signaling cascade, which stimulates EMT and the production of collagen [45].

There is growing experimental evidence that hypoxia via HIF promotes EMT. This occurs through i) modulation of activity of EMT inducers or their receptors (e.g. TGF-β1; c-met proto-oncogene [28]), ii) regulation of expression levels and activity of EMT-associated signaling molecules and downstream effectors (e.g. Notch and β-catenin [46,47]), and iii) via regulation of expression and activity levels of EMT-inducing transcriptional repressors, such as snail (SNAI1) [48]. In the context of CKD, renal tubular epithelial cells loose apico-basal polarity, become motile and acquire a mesenchymal phenotype. It has been proposed that they are able to migrate into the interstitium, where they together with resident cells would produce ECM as myofibroblasts [24]. Irrespective of cellular origin, the accumulation of myofibroblasts closely correlates with the degree of interstitial damage and the risk of disease progression. Earlier in vivo studies using genetically tagged renal epithelial cells have suggested that up to 36% of interstitial myofibroblasts are EMT-derived, whereas approximately 15% stem from bone marrow, with the remainder being of resident fibroblast origin [21]. The degree to which EMT contributes to the renal myofibroblast pool, however, is intensely debated and remains unclear [49]. More recent studies indicate that pericytes [50] and/or endothelial cells [51] are major sources of ECM-producing myofibroblasts.

HIF-1 induction of LOX and LOXL-2 has been shown to promote migration in primary renal epithelial, and in breast and cervical cancer cells, which was associated with decreased E-cadherin expression [5,25]. Although initially identified by their ability to crosslink collagen and elastin fibers, LOX and LOXL proteins also carry out intracellular functions and display a range of biological activities that extend beyond ECM cross-linkage [26]. In a recent report Peinado et al. demonstrated that LOXL2 and LOXL3 have the potential to regulate EMT by stabilizing and promoting the activity of transcriptional repressor SNAI1 [52].

**Figure 5:**
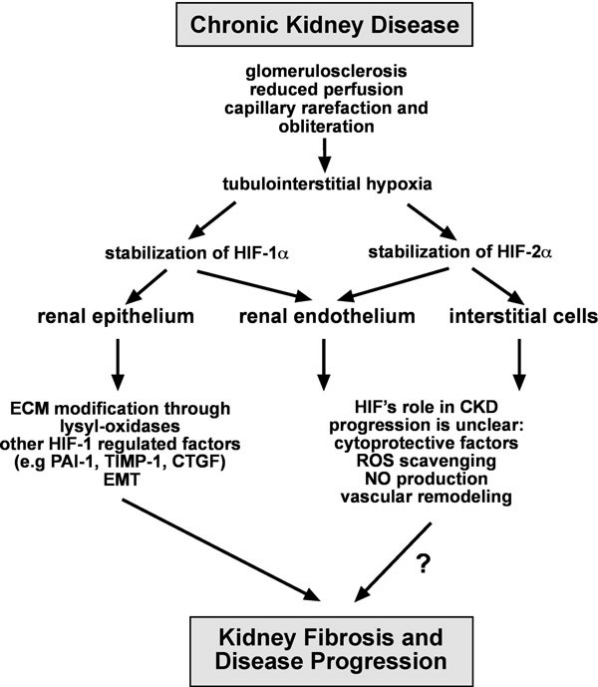
**Overview of HIF signaling in CKD**. Shown is a summary of potential mechanisms by which epithelial HIF-1 could act as a profibrotic transcription factor. These include the regulation of ECM production and processing through matrix modifying factors and enzymes, such as CTGF, PAI-1, TIMP-1 and MMPs, and the modulation of EMT triggering pathways. The role of non-epithelial HIF in the pathogenesis of renal fibrogenesis is not known. **Abb.: **CTGF, connective tissue growth factor; ECM, extra-cellular matrix; EMT, epithelial to mesenchymal transition; NO, nitric oxide; PAI-1, plasminogen activator inhibitor 1; ROS, reactive oxygen species; TIMP-1, tissue-inhibitor of metalloproteinase 1.

Our finding that HIF activation in epithelial cells promotes renal fibrosis has immediate clinical implications, as it encourages therapies that aim at improving tissue oxygenation to retard disease progression. Because HIF regulates multiple biological processes, which include erythropoiesis and iron metabolism, its systemic pharmacological inhibition is not desirable. Certain HIF regulated proteins, however, may represent better therapeutic targets to slow progression of CKD. Pharmacological inihibition of lysyl oxidases phenocopied the effects of genetic HIF-1 inactivation on cell motility and fibrogenesis, suggesting that lysyl oxidases are important contributors to the pathogenesis of renal fibrosis. In keeping with this notion, increased *LOXL2 *expression was found in renal biopsy tissues from patients with CKD underscoring its relevance to the pathogenesis of CKD irrespective of etiology (Figure 5). Whether pharmacological inhibition of lysyl oxidases is feasible in clinical practice to slow progression of CKD certainly warrants further investigation.

## Competing interests

The author declares that he has no competing interests.
